# Clinical Pearls - how my patients taught me: The fainting lark symptom

**DOI:** 10.1186/s40734-016-0045-8

**Published:** 2016-11-02

**Authors:** A. Kuiper, M.E. van Egmond, M.P.M. Harms, M.D. Oosterhoff, B. van Harten, D.A. Sival, T.J. de Koning, M.A.J. Tijssen

**Affiliations:** 1 Department of Neurology, University of Groningen, University Medical Center Groningen, Hanzeplein 1, PO box 30.001, 9700RB Groningen, The Netherlands; 2 Department of Internal Medicine, University of Groningen, University Medical Center Groningen, Groningen, The Netherlands; 3 Jonx Department of Youth Mental Health, Lentis Psychiatric Institute, Groningen, The Netherlands; 4 Department of Neurology, Medical Centre Leeuwarden, Leeuwarden, The Netherlands; 5 Department of Pediatrics, University of Groningen, University Medical Center Groningen, Groningen, The Netherlands

**Keywords:** Respiratory stereotypies, Compulsive Valsalva, Stretch syncope

## Abstract

**Background:**

Compulsive movements, complex tics and stereotypies are frequent, especially among patients with autism or psychomotor retardation. These movements can be difficult to characterize and can mimic other conditions like epileptic seizures or paroxysmal dystonia, particularly when abnormal breathing and cerebral hypoxia are induced.

**Case presentation:**

We describe an 18-year-old patient with Asperger syndrome who presented with attacks of tonic posturing of the trunk and neck. The attacks consisted of self-induced stereotypic stretching of the neck combined with a compulsive Valsalva-like maneuver. This induced cerebral hypoperfusion and subsequently dysautonomia and some involuntary movements of the arms.

**Conclusion:**

This patient suffered from a complex tic with compulsive respiratory stereotypies. His symptoms contain aspects of a phenomenon described in early literature as ‘the fainting lark’.

**Electronic supplementary material:**

The online version of this article (doi:10.1186/s40734-016-0045-8) contains supplementary material, which is available to authorized users.

## Background

Paroxysmal abnormal movements can be a challenging complaint for clinicians. Often the question raises whether the attacks are epileptic in nature; the first concern is to make the distinction between epileptic and non-epileptic attacks.

Although epilepsy is often thought of first, non-epileptic attacks are very frequent in children and adolescents [1]. Non-epileptic attacks comprise a wide spectrum of disorders, including paroxysmal movement disorders, functional (psychogenic) attacks, Sandifer syndrome, cardiac events and behavioral events. Stereotypies, tics and compulsive movements are frequent, especially in patients with psychomotor retardation or autistic features. Sometimes these movements affect the breathing pattern [2, 3]. In a case series of eight patients with autistic features two types of compulsive respiratory stereotypies were recognized: simple apneas, mainly seen in patients with severe psychomotor retardation; and forced expirations against a closed glottis (Valsalva maneuver), mainly seen in autistic patients with less severe mental retardation [4]. This Valsalva maneuver is also part of a phenomenon known as the fainting lark, a game of school children to voluntarily induce a syncope [5].

The aim of this paper is to present a patient with a complex tic with compulsive respiratory stereotypies, containing aspects of the fainting lark maneuver.

## Case presentation

An 18-year-old man presented to our movement disorder clinic with attacks of tonic posturing of the trunk, neck and upper extremities. The attacks lasted a few seconds and were often provoked by rising from a chair. During an attack autonomic features were present, including pallor, nausea and sweating. The patient told that during an attack he felt like he was going to faint, but he never completely lost consciousness. In between attacks the neurological examination was completely normal. The attacks had started since the age of 14 years and had become more frequent over time, now occurring up to 7 times a day. This had resulted in considerable disruption of daily life, as the attacks significantly hampered his school performance and social activities. The past medical history revealed Asperger syndrome with normal cognition and simple motor tics in early childhood.

During the outpatient clinic visit and later during electrophysiology and autonomic function tests, we observed several attacks (see Additional file 1). All events started as the patient arose from a seated position. He rotated his head to the left with retroflexion. The trunk is subsequently extended and both arms showed some dystonic posturing and involuntary movements. The patient became pale and started to perspire. After several seconds the attack stopped abruptly, and the patient was able to sit down again. He did not proceed into a syncope, but indicated clouding of consciousness during the attacks.

The initial differential diagnosis included epileptic seizures, paroxysmal kinesiogenic dyskinesia (PKD), benign paroxysmal dystonia and Sandifer’s syndrome. An EEG during the attacks showed some background slowing but no epileptic activity. Mutation analyses of the PRRT2 gene (PKD) and CACNA1A gene (benign paroxysmal dystonia) were negative. Because of the prominent dysautonomia during the attacks, we performed autonomic function tests of blood pressure regulation. These tests, which included a tilt table test, showed no signs of autonomic dysfunction.

During these tests, however, it became clear that the patient could intentionally provoke the attacks. On closer observation, we noticed that the attacks were consistently triggered by taking deep breaths, and during the attack there were no respiratory excursions. It appeared that the combination of stretching of the neck and performing a Valsalva-like maneuver resulted in cerebral hypoxia. The observed attacks were all very similar, illustrating the stereotyped character.


Video: The video shows two attacks, recorded on two different outpatient clinic visits. By showing two attacks, the stereotype character is illustrated. The attacks start with rising from a seated position, while extending and rotating the neck. The patient is pale and starts to perspire, while there is abnormal posturing of the trunk and some involuntary movements of the arms and hands occur. After several seconds the attack stops quite abruptly, and he is able to function normally again. When the video is closely watched, it can be seen that both attacks start with taking a deep breath and that during the attacks there are no respiratory excursions (MPG 27200 kb)


The patient later endorsed a premonitory urge and brief ability to voluntary suppress an attack. There was no sense of relief afterwards. Based on these characteristics, and since the movements were self-induced, compulsive and stereotyped, the phenomenology can be described as a complex tic.

The patient was already treated by a psychiatrist, but psychotherapy and antipsychotic drugs had been insufficiently effective to improve his symptoms. Therefore, treatment with botulinum toxin was initiated in our patient to interrupt the tic-like extension of the neck and subsequent cerebral hypoperfusion. Over the past 2 years he has been receiving quarterly injections of 15–25 units of botulinum toxin in multiple muscles of the neck (left longissimus, semispinalis, levator scapulae, obliquus, splenius capitis and trapezius muscles; and right longissimus muscle). This has resulted in a strong decrease in both the frequency and severity of attacks, now only occurring incidental. He is again able to normally participate in study activities.

## Conclusions

We described a case of a young male patient who suffered from self-induced, compulsive respiratory stereotypies or complex tics. The attacks in this patient include features of a phenomenon described in earlier literature as ‘the fainting lark’ [5].

The fainting lark is known as a game played by school and college students. During this game a person bends his knees, takes several deep breaths, and then suddenly stands up while performing a forced expiration against a closed glottis (Valsalva manoeuver). This technique combines the effects of acute orthostatic stress, straining and hyperventilation and results in acute cerebral hypoxia that can lead to syncope [6]. Besides a game of school children, Valsalva manoeuvers inducing a syncope have been recognized as a compulsive behaviour in children and adolescents with autism spectrum disorders [2].

In addition to dysautonomia and syncope, compulsive breath holding and subsequent cerebral hypoxia can induce involuntary movements. These hypoxia-inducing maneuvers can result in a typical extension of the upper limbs with elevation and abduction. The transient cerebral hypoperfusion is also the cause of EEG background slowing and can even result in epileptic seizures [3].

It can be challenging to distinguish compulsive respiratory stereotypies or complex tics from other causes of paroxysmal involuntary movements. In our patient repeated close observation of the attacks eventually led to the diagnosis, where the aberrant breathing pattern and compulsive, stereotyped nature of the attacks were essential clues. Despite early descriptions of respiratory stereotypies like the fainting lark phenomenon, there has been little attention to this in recent literature. Nevertheless, it is important to recognize and distinguish this etiology from other conditions in order to guide treatment. As demonstrated in this case, botulinum toxin injections can be useful to interrupt a complex tic.

## Abbreviations

EEG: Electroencephalogram
PKD: Paroxysmal kinesiogenic dyskinesia
